# Representation of Women in the Leadership Structure of the US Health Care System

**DOI:** 10.1001/jamanetworkopen.2021.36358

**Published:** 2021-11-29

**Authors:** Bismarck C. Odei, Crystal Seldon, Melanie Fernandez, Michael K. Rooney, Junu Bae, Jason Acheampong, Awad Ahmed

**Affiliations:** 1 James Cancer Center, The Ohio State University, Columbus; 2 Sylvester Comprehensive Cancer Center, University of Miami, Miami, Florida; 3 Northeast Ohio Medical University, Rootstown; 4 MD Anderson Cancer Center, Houston, Texas; 5 The Ohio State University School of Medicine, Columbus; 6 Morehouse School of Medicine, Atlanta, Georgia; 7 MultiCare Tacoma General Hospital, Tacoma, Washington

## Abstract

This cross-sectional study evaluates the gender distribution among chief executives and senior leaders at US health care organizations.

## Introduction

The US health care system serves a heterogeneous population with varied health care needs,^1^ which underscores the importance of diverse perspectives in health care leadership. However, few contemporary studies have assessed gender diversity in the leadership structure of organizations (health systems and health insurance groups) that form the US health care system. Consequently, this study evaluated the representation of women among the highest-ranking executives in the US health care system.

## Methods

This cross-sectional study was deemed exempt from review by The Ohio State University Institutional Review Board, which waived the informed consent requirement because the data were publicly available. We followed the Strengthening the Reporting of Observational Studies in Epidemiology (STROBE) reporting guideline.

For this analysis, we identified the following health care organizations: health systems with a minimum of 5 affiliated hospitals,^2^ health insurance groups with at least 0.09% of the US health insurance market share,^3^ and the US Department of Health and Human Services. We collected information on executives from each organization’s website. Between April 1 and May 31, 2021, we collected data on the gender of members of the senior executive leadership teams and/or boards of directors (BODs) of the selected organizations. Each executive was assigned to binary categories of gender (man or woman), and gender was identified by reviewing a combination of gender identifiers, names, and corresponding photographs. Organizations without available online data on their executives and individuals for whom gender could not be determined were excluded from the study.

Descriptive statistics were used to define the distributions of continuous variables. One-sided binomial testing was used to compare the gender representation within individual positions, with the null hypothesis that gender distribution was equally men and women at all positions (p = 0.5; q = 0.5). Multiple binary logistic regression modeling was performed to identify the factors associated with having a woman as chief executive officer (CEO) using all of the variables shown in the Table.

**Table.** Multiple Binary Logistic Regression Model to Identify Factors Independently Associated With the Presence of a Woman as Chief Executive Officer

Group	Variable	OR (5%-95% CI)	*P* value
Health systems	No. of physicians in health care system	0.99 (0.99-1.00)	.93
	No. of hospitals in health care system	0.97 (0.89-1.01)	.26
	Size of BODs	0.96 (0.86-1.05)	.40
	Proportion of women on BODs	1.09 (1.01-1.18)	.03
	Presence of a woman as chairperson on BODs	0.33 (0.06-1.34)	.15
	No. of senior health system executives	0.96 (0.89-1.02)	.24
	Proportion of women among health system executives	1.06 (1.02-1.11)	.008
Health insurance groups	Size of BODs	1.01 (0.84-1.18)	.89
	Proportion of women on BODs	0.99 (0.91-1.05)	.66
	Presence of a woman as chairperson on BODs	4.29 (0.71-29.2)	.12
	No. of senior health insurance executives	0.79 (0.60-1.02)	.05
	Proportion of women among health insurance executives	1.06 (1.01-1.12)	.02

Abbreviations: BODs, boards of directors; OR, odds ratio.

Statistical analyses were conducted using R, version 4.0.3 (R Foundation for Statistical Computing). *P* < .05 indicated statistical significance. We used unpaired binomial testing, with a 1-sided *P* value, and binary logistic regression, with a 2-sided *P* value.

## Results

A total of 3911 senior executives (2608 [66.7%] from health systems, and 1303 [33.3%] from health insurance groups) and 3462 BODs (2319 [67.0%] from health systems, and 1143 [33.0%] from health insurance groups) were examined, representing 161 health systems and 108 health insurance groups. We also assessed 31 leadership positions within the US Department of Health and Human Services. Of these executives, 13 were men (41.9%) and 18 were women (58.1%).

The median (IQR) size of the executive teams was 14 (11-20) members for health systems and 11 (9-15) members for health insurance groups. Among BODs, the median (IQR) size was 15 (11-19) members for health systems and 13 (10-14) members for health insurance groups.

The proportion of BOD chairpersons who were women was 17.5% in health systems and 21.3% in health insurance groups. Only 15.3% of the CEO roles in health systems and only 15.8% of the CEO positions in health insurance groups were held by women (Figure, A). Among BODs and senior executive teams in both health systems and health insurance groups, we found that approximately 20% to 50% of leadership positions were filled by women (Figure, B and C).

**Figure.**
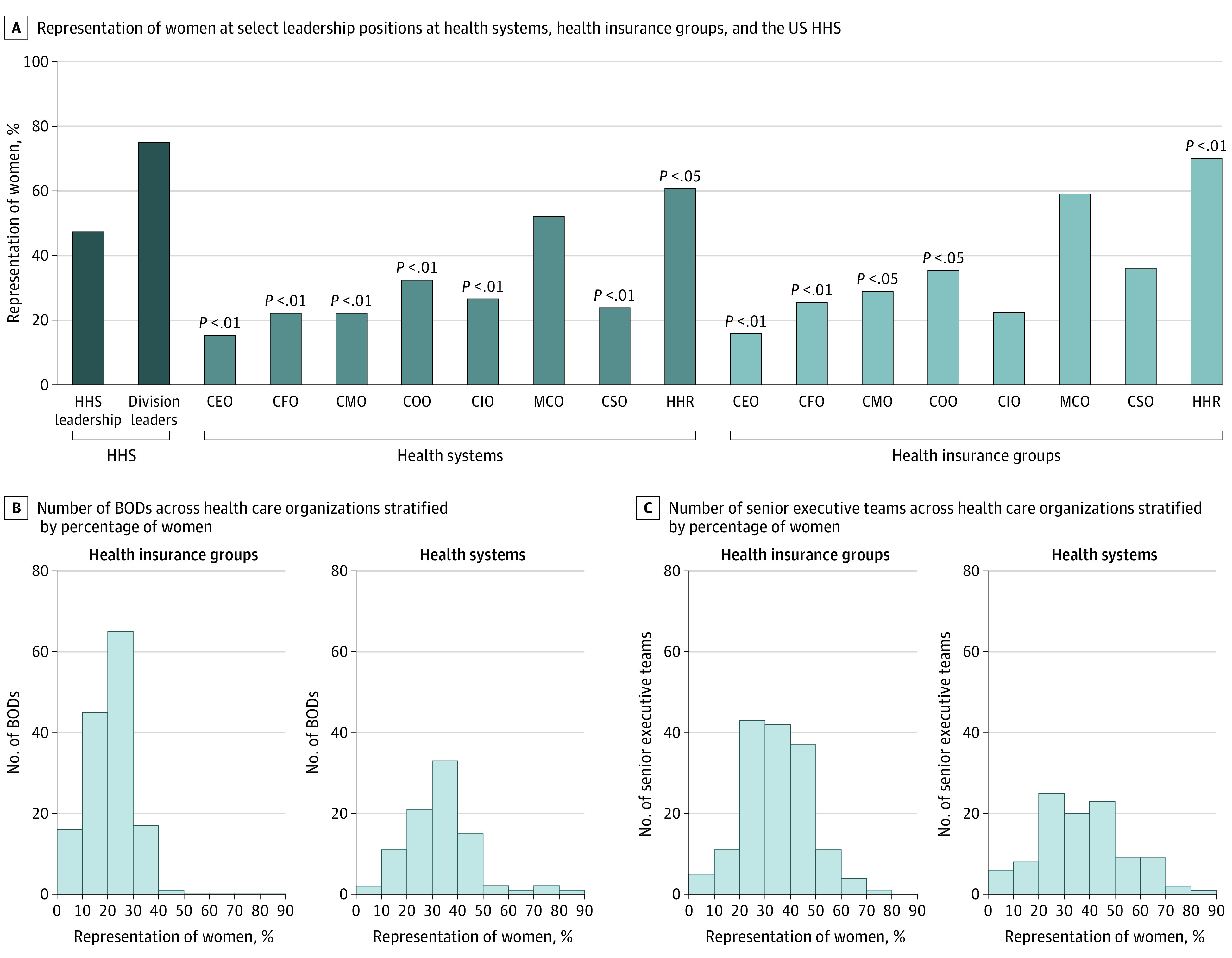
Representation of Women in Executive Positions in the US Health Care System BOD indicates board of directors; CEO, chief executive officer; CFO, chief financial officer; CIO, chief information officer; CMO, chief medical officer; COO, chief operating officer; CSO, chief strategy officer; HHR, human resources officer; HHS, US Department of Health and Human Services; MCO, marketing/communications officer.

In the health systems, a woman as CEO was associated with a higher proportion of women either on the BODs (odds ratio [OR], 1.09; 5%-95% CI, 1.01-1.18; *P* = .03) or in senior executive positions (OR, 1.06; 5%-95% CI, 1.02-1.11; *P* = .008). Similarly, a higher proportion of women on senior executive teams of health insurance groups was associated with increased representation of women as CEOs (OR, 1.06; 5%-95% CI, 1.01-1.12; *P* = .02).

## Discussion

Although women currently represent a slight majority of the US population and a large majority of the US health care workforce,^4^ they are generally underrepresented on leadership teams, which likely diminishes their role in policy decisions that affect population and women’s health. In addition, a recent study reported an association between gender diversity in organizational leadership and improved organizational performance,^5^ suggesting the loss of cognitive capital with the underrepresentation of women on executive teams.

Women held only approximately 15% of the CEO positions in health care organizations. Members of the BOD, whose primary responsibility includes choosing a CEO, appeared to have a role in increasing the representation of women as CEOs in health systems when the BODs had a more gender-diverse composition. It also appeared that organizations with senior leadership with gender diversity were more likely to have a woman as CEO.

In this study, the factors associated with the representation of women may have been limited in scope. Furthermore, assigning gender to binary categories was a limitation. The findings support the increased prioritization of gender diversity^6^ at all hierarchical areas in the US health care system.

## References

[1] Mokdad AH, Ballestros K, Echko M, ; US Burden of Disease Collaborators. The state of US health, 1990-2016: burden of diseases, injuries, and risk factors among US states. JAMA. 2018;319(14):1444-1472. doi:10.1001/jama.2018.0158 29634829 PMC5933332

[2] Agency for Healthcare Research and Quality. Compendium of U.S. health systems, 2018. Accessed September 20, 2021. https://www.ahrq.gov/chsp/data-resources/compendium-2018.html

[3] National Association of Insurance Commissioners. 2020 Market share reports for the top 125 accident and health insurance groups and companies by state and countrywide. Accessed September 20, 2021. https://content.naic.org/sites/default/files/publication-msr-hb-accident-health.pdf

[4] Cheeseman Day J, Christnacht C. Women hold 76% of all health care jobs, gaining in higher-paying occupations. August 14, 2019. Accessed September 20, 2021. https://www.census.gov/library/stories/2019/08/your-health-care-in-womens-hands.html

[5] Dixon-Fyle S, Dolan K, Hunt V, Prince S. Diversity wins: how inclusion matters. May 19, 2020. Accessed September 20, 2021. https://www.mckinsey.com/featured-insights/diversity-and-inclusion/diversity-wins-how-inclusion-matters

[6] Women in Global Health. Challenging power and privilege for gender equity in health. Accessed September 20, 2021. https://www.womeningh.org

